# Addressing anti-Black racism within public health in North America: a scoping review

**DOI:** 10.1186/s12939-024-02124-4

**Published:** 2024-06-28

**Authors:** Lucina Rakotovao, Michelle Simeoni, Caroline Bennett-AbuAyyash, Taheera Walji, Samiya Abdi

**Affiliations:** 1 Public Health Ontario, 661 University Avenue, Suite 1701, Toronto, ON M5G 1M1 Canada; 2 Public Health Agency of Canada, 180 Queen St W, 11th Floor, Toronto, ON M5V 3X3 Canada; 3 Dalla Lana School of Public Health, University of Toronto, Toronto, ON M5T 3M7 Canada; 4 Ontario Brain Institute, 1 Richmond Street West, Suite 400, Toronto, ON M5H 3W4 Canada; 5 Black Health Education Collaborative, 720 Bathurst Street, Suite 420, Toronto, ON M5S 2R4 Canada

**Keywords:** COVID-19, Equity, Anti-black racism, Public health

## Abstract

**Objectives:**

The syndemic that is COVID-19 and the disproportionate policing of Black communities have recently generated mass social consciousness of the anti-Black racism (ABR) pervading health, social, and cultural institutions. However, little is known about the implementation of public health measures addressing ABR in an evolving pandemic context. The objective of this scoping review is to provide an overview of public health initiatives undertaken to address ABR across North American jurisdictions between December 2019 and June 2022.

**Methods:**

A search for public health initiatives was conducted in June 2021 across MEDLINE, Ovid Embase, EBSChost, CINAHL, SocINDEX, and Google.ca. Included initiatives were those focussing on Black, African diasporic, or African American communities in the North American context. Community-led action, as well as initiatives in primary healthcare care, academic journals, and those broadly focused on racialized communities, were excluded from this review.

**Synthesis:**

Seventy-five articles were included in this review, suggesting that ABR emerged as a public health priority. Strategies and action plans to address structural ABR were the most common types of initiatives observed (*n* = 21), followed by programs or interventions (*n* = 16), budget allocations or investments (*n* = 8), task forces (*n* = 7), guidance and recommendations for organizational capacity (*n* = 8), action-oriented declarations of ABR as a public health crisis (*n* = 8), and legislation and mandates (*n* = 7). Initiatives were largely cross-cutting of two or more socioeconomic themes (*n* = 23), while organizational change was also common (*n* = 16). Gaps in the current literature include a lack of community participation and outcome measurement for actions identified, which limit institutional accountability to communities of interest.

**Conclusion:**

This research provides insights on public health accountability to social justice. This research outlines activities in upstream interventions, organizational transformation, and resource allocation in shaping anti-racist change, and require evaluation and input from those whom initiatives are intended to serve.

## Background

The COVID-19 pandemic has unequivocally highlighted and exacerbated race-based health inequities on an international scale. Considering the impact of media, issue visibility, and public pressure on policymaking, COVID-19 has equally precipitated a mass critical consciousness for racial injustice as a public health crisis [1, 2]. In particular, the onslaught of anti-Black police violence publicized in 2020 – including the murders of George Floyd, Ahmaud Arbery, D’Andre Campbell, Regis Korchinski-Paquet, and Breonna Taylor – has catapulted widespread acknowledgement of the Black Lives Matter movement and calls for justice for marginalized lives [3]. Combined with the finding that systemic anti-Black racism (ABR) translates into detrimental health outcomes for Black communities [1], including disproportionate higher rates of COVID-19 infection [4], there has been growing awareness across sectors that systemic racism and intersectional violence rooted in classism, ableism, sexism, heterosexism, and more, create and sustain Black health disparities [5, 6]. This has resulted in pervasive calls to action in public health to ethically disaggregate data to elucidate Black-specific inequities; integrate institutional accountability measures in equity, diversity, and inclusion (EDI); and include anti-racism and historical harms in the ongoing implementation of COVID-19 measures, among others [7].

The physical and psychological harms Black communities accrue over a lifespan are grounded in collective historical traumas such as slavery, genocide, displacement, and ongoing state-sanctioned violence like police surveillance and brutality [8–10]. Further, the recurring devaluation of Black lives depicted in media and polarizing societal mood (e.g., rising white supremacy) perpetuates community harm through chronic race-based traumatic stress [8, 11, 12]. This cycle of violence warrants urgent action from public health institutions. However, little is known about the extent to which measures addressing ABR have been implemented since calls for racial justice punctuated an evolving pandemic context. Referenced measures include any directed internal or external programs or interventions, actionable declarations or statements, budget allocations and investments, guidance and recommendations for organizational change, task forces, strategies or action plans, or legislative change.

### Objectives and scope

The aim of this review is to elucidate the frequency and breadth of initiatives undertaken by North American public health institutions, spanning a wide range of settings including governmental and non-governmental agencies, regional health authorities, and non-profit organizations. The authors and Public Health Ontario (PHO) Library Services Specialists sought literature from December 2019 to June 2022 in alignment with the onset of the COVID-19 pandemic and concordant rise of ABR reported transnationally [13–15]. Geographically, literature was limited to Canada and the United States (US) to increase the relevancy and transferability of results to a Canadian public health context. This geographic scope equally highlights the degree of Canada-specific public health performance and accountability to values of health equity and social justice.

The present scoping review was thus guided by the following question: How have entities with public health mandates, across jurisdictions, actioned calls to dismantle ABR within their institutions and in the interest of public good since the onset of the COVID-19 pandemic?

This scoping review is informed by the central tenets of Critical Race Theory (CRT), which acknowledge race as social construct rather than a biological factor and that racism is intentionally and systemically imbedded across sectors such as health care [16, 17]. A CRT lens further attempts to mobilize transformative action against injustices beyond simple awareness [16, 17]. With CRT as a guiding framework, the authors thus acknowledge how the establishment of health and social institutions were/are premised on maintaining racial and class hierarchies and thus, radicality within these institutions may exist on a spectrum. In other words, the language of “EDI” in institutional settings should not be conflated with radical modes of anti-racist organizing, which use liberatory, anti-oppressive praxes to enable the complete transformation of systems not originally designed for those at the margins [17]. While this research aims to equip public health institutions with a greater evidence base of practices to address ABR, it ultimately aims to encourage broad implementation of health justice action, which as a movement seeks to recognize and build power of individuals and communities affected by health inequities and spur transformational change of systems that drive those inequities [18]. This transformational change transcends institutional EDI objectives that are constrained by corporate governance frameworks premised on various forms of exclusion or interests. The authors further acknowledge the heterogeneity of Black communities and emphasize the importance of centering community context in developing initiatives, based on locality, history, and community needs and recommendations.

For the purposes of this research, “Black” is used as an aggregate term to describe the African diaspora and communities of Caribbean descent, including descendants of enslaved or free peoples with no immigration histories [19]. This nuance is acknowledged with recognition that Black existence is differentially experienced across heterogeneous social contexts and identities, and that membership in a community can be highly complex rather than monolithic [20].

## Methods

Scoping reviews are a type of comprehensive review and knowledge synthesis conducted to understand the state of the literature on a novel or emerging topic and to identify research gaps in the existing evidence [21, 22]. A scoping review was selected to both identify knowledge gaps and implications for decision making and setting research agendas on dismantling ABR in the public health sector in North America [23]. The methodology adhered to the accepted framework for conducting scoping studies by Arksey & O’Malley (2005) and included the following stages: identifying the research question; identifying relevant studies; study selection; data extraction; collating, summarizing and reporting the results; and consulting with PHO staff and reviewers with subject matter expertise on manuscript presentation and coherence [22].

Both grey and peer-reviewed literature were investigated for this review. For the purposes of this review, grey literature includes the array of evidence produced outside of commercial publishing organizations and distribution channels [24]. PHO Library Services Specialists developed and executed the peer-reviewed literature searches, after which the authors conducted both abstract and in-depth screenings of identified records. Literature searches were conducted in MEDLINE, Ovid Embase, EBSCOhost CINAHL, and SocINDEX. PHO Library Services Specialists also provided guidance with grey literature search strategies, which were subsequently conducted by the authors. In addition to Google.ca, grey literature searches were conducted in the following custom search engines: Ontario Public Health Units; Canadian Health Departments and Agencies; US State Government Websites; and US.Gov/.Org/.Edu domains. The first 100 results were screened for relevant content (per search string), as relevance decreases after the first 50–100 results. Searches were conducted using four queries of search terms including but not limited to: Anti-Black, Black, African diaspora, African descent, or African-American; racism, discrimination, injustice, inequality, inequity, disparities, or oppression; and “public health” action, policy, advocacy, measures, programs, initiatives, declaration, statement, practice, support, or strategy. Both peer-reviewed and grey literature search strategies used similar search terms, including relevant Medical Subject Headings (MeSH) where appropriate. The full search strategies and selected terms are available upon request.

Two levels of screening (title and abstract and full-text) were independently conducted by two reviewers from PHO. Included literature underwent a full-text review for extraction by three independent reviewers by theme (e.g., social determinant of health, organizational change) and type (e.g., task force, legislation, fiscal investment, etc.). Results were tracked and synthesized in an Excel log. Authors met regularly to agree on themes for organizing the data for inclusion in the results, particularly to discuss any records containing ambiguous or misaligned objectives with the chosen inclusion criteria. In addition to screening records through a CRT lens, reviewers also used a Black health justice lens, whereby consensus for inclusion was sought based on records’ explicit foci on power-building and transformational change for Black populations with a liberatory, reparative, or transformative aim. The authors chose to focus on implemented, supported, or mandated anti-racism action from any level of government, health agency, or organization to identify a repository of feasible interventions for implementation across public health contexts. In this context, authors selected records with “actionable” initiatives that were either specific, measurable, achievable, realistic, or time-bound (SMART). Literature considered outside of scope for this review included non-targeted population health initiatives (e.g., broad diversity or equity initiatives for racialized communities), community-led action, initiatives in primary healthcare care, education settings, or knowledge production institutions (e.g., academic journals), and public health statements that did not include actionable commitments.

Results are presented based on action types: strategies and action plans to address structural ABR, programs and interventions, budget allocations or investments, task forces, guidance and recommendations for organizational capacity, action-oriented declarations of ABR as a public health crisis, and legislation and mandates.

## Results

### Summary of findings

**Fig. 1:**
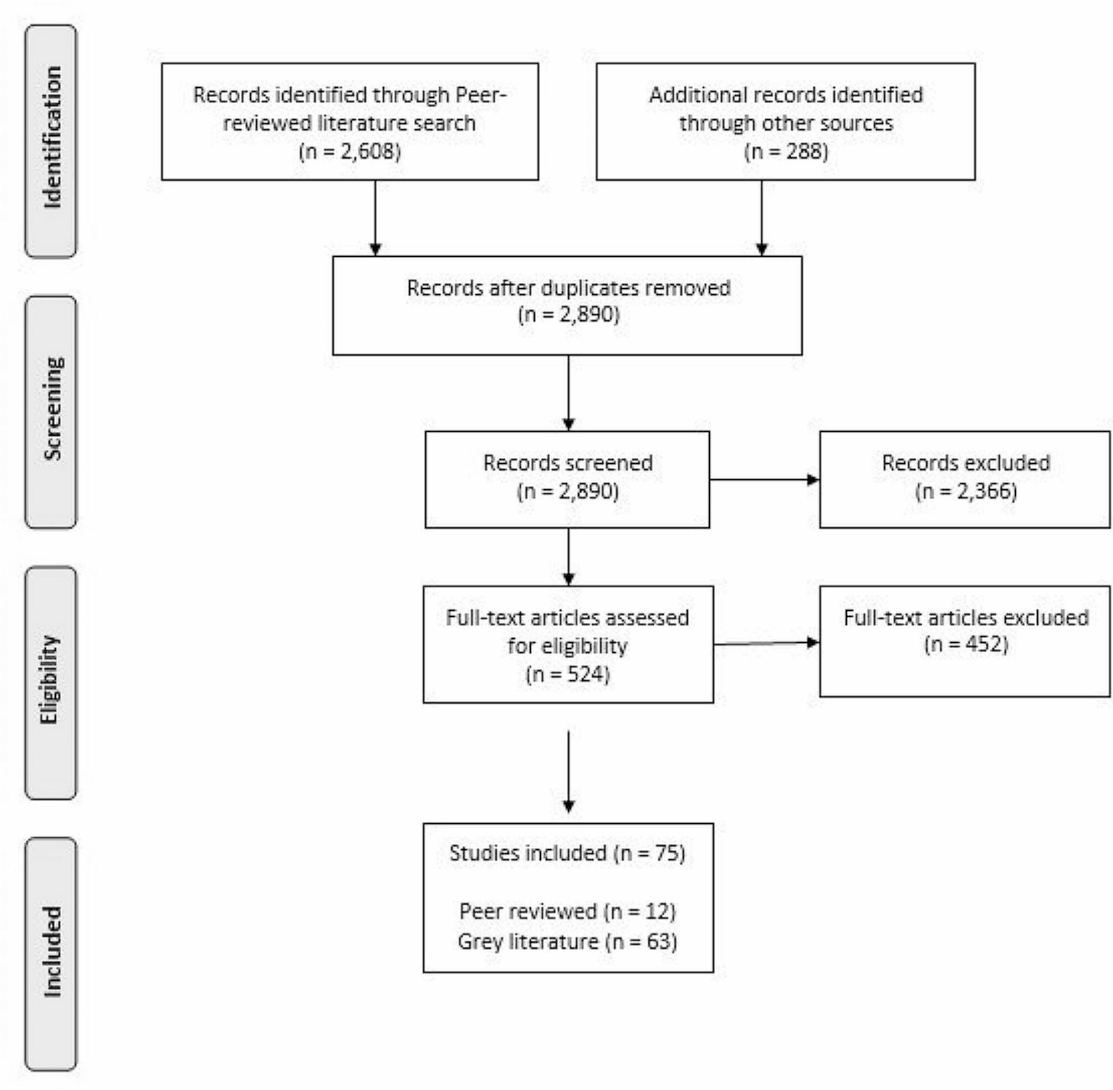
PRISMA flowchart outlining article selection process [25]

The peer-reviewed search yielded 2,608 articles. After removing duplicates, 2,602 articles were screened through title and abstract screening and 288 articles advanced to full-text screening. Twelve peer-reviewed articles were included. The grey literature search identified 288 records, of which 63 articles met the inclusion criteria following full text screening. Seventy-five articles that met the inclusion criteria were identified between grey and peer-reviewed literature. The adapted PRISMA flowchart in Fig. 1 outlines the study selection process [25].

Of these 75 included results, 35 were of a Canadian jurisdiction, 37 of an American jurisdiction, and three of Pan-American relevance. Included literature was published from December 2019 to June 2022, of North American origin, stemming directly from public health institutions or of relevance to public health, and with the explicit mention of Black communities.

Each type of public health action is further summarized by key theme or area of foci in Table 1, which are often co-occurring:

- Community development/programs/services.
- Data disaggregation.
- Education.
- Food security and sovereignty.
- Health.
- Income/Poverty/Employment.
- Organizational change (e.g., hiring, training).
- Policing and justice.
- Youth.

**Table 1:** Frequency and distribution of social determinants of health across areas of focus

	Actionable declaration or statement	Budget allocation or investment	Guidance, recommendations on organizational capacity strengthening	Legislation and mandates	Program or intervention	Strategy and action plan	Task force	Total
Education	3				1	4		8
Employment, income, &/or poverty	1		1			1		3
Food security and sovereignty						2		2
Policing and justice	4		1	3	1	4		13
Community development	4	5	1	2	1	18		21
Health	8	5	1	4	12	15	2	47
Organizational change	3			2	1	7		13
Data disaggregation				1	2	7	1	11
Youth		5	1			4		10

*The City of Toronto strategy for confronting anti-Black racism includes multiple associated documents (original documents, updates, appendices). These have been coded as one item in the table above

**Fig. 2:**
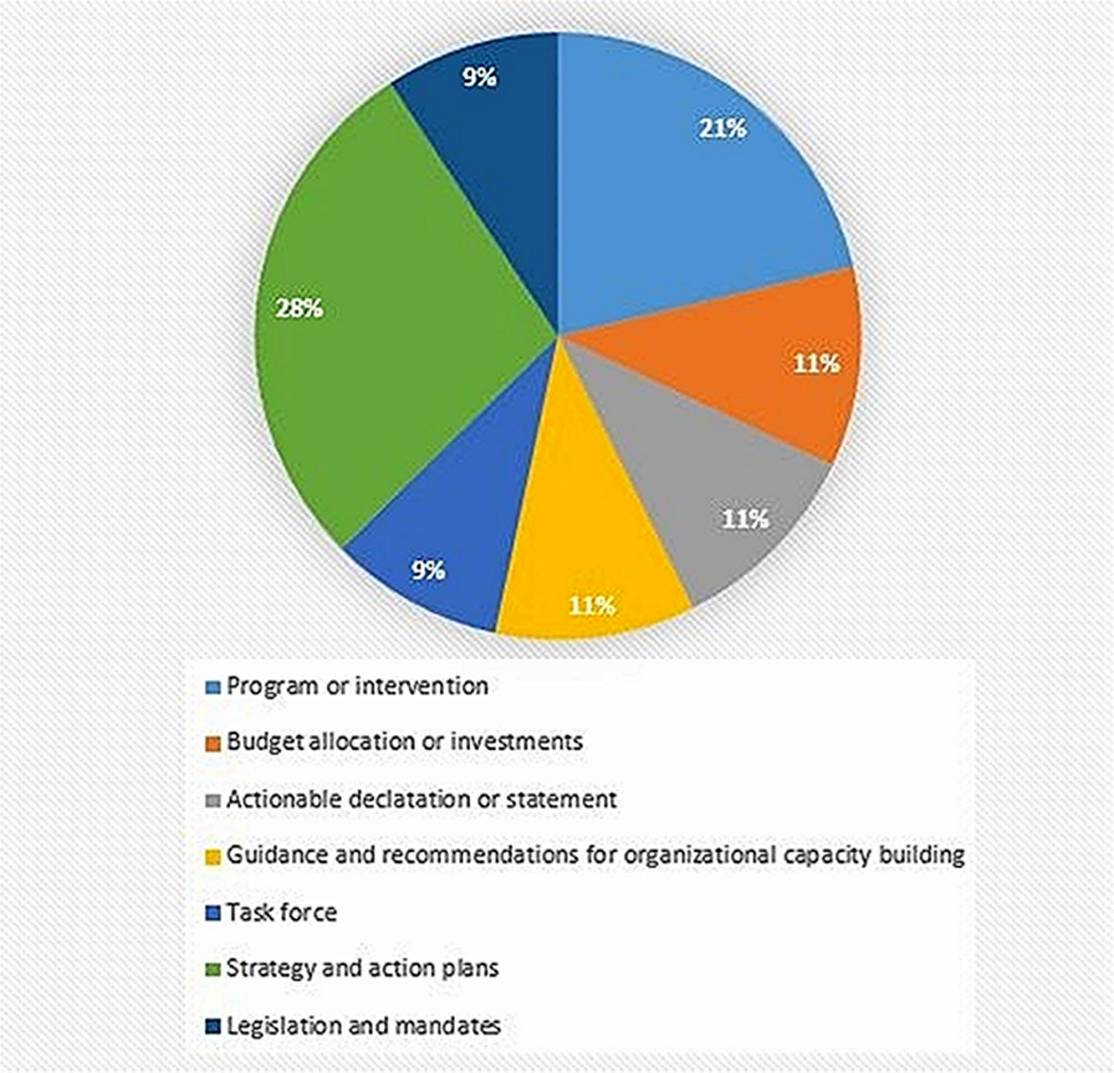
Summary of action types found

Strategies and action plans (28%) and programs and interventions (21%) to address structural ABR were the most common types of action observed in the present review, followed by budget allocation or investments (11%); task forces (11%); organizational capacity strengthening (11%); declarations of ABR as a public health crisis (11%); and legislative change (9%) (Fig. 2).

### Program or intervention

A total of 16 records included public health interventions and programs (see Table 1). Across all jurisdictions, interventions possessed an explicit focus on improving access to or the quality of health services for Black populations.

All interventions identified in the national or transnational context held a COVID-19 focus. In a Pan-American context, the Pan American Health Organization (PAHO) urgently called for racial equality measures in the transnational COVID-19 response, and committed to prioritizing populations of African descent through targeted data collection programs and initiatives to improve access to health services [26]. In the US, three initiatives characterized national efforts to address racism and disparities in Black health related to COVID-19 prevention and treatment [27, 28]. The National Academy of Medicine, for example, led a public health campaign to increase vaccine uptake by Black Americans using a New York Times op-ed and promotional YouTube video [27]. The US Department of Health and Human Services supported community-led, language diverse COVID-19 communication programs, a Provider Relief Fund, debt protection measures for uninsured Black Americans, and data disaggregation approaches with the Centers for Disease Control and Prevention [29]. The Boston University Center for Antiracist Research also initiated a National COVID-19 Racial Data Tracker that disaggregated COVID-19 statistics by race and ethnicity [28].

Using COVID-19 as an opportunity for action, jurisdictions like Boston [30], Arkansas [31], California [32], New Orleans [33], and the State of Wisconsin [34] indicated responsiveness to Black disparities in COVID-19 by implementing needs-based mobile testing, vaccine access, and outreach throughout 2020 and 2021. The eleven sites of California’s statewide alliance STOP COVID-19 CA, for example, leveraged long standing community partnerships and engagement to better understand concerns, misinformation, and address racial/ethnic inequities in vaccine hesitancy and uptake in their communications [32]. LA County also implemented equity-informed strategies like population-specific testing, vaccination, and therapeutics [35]. New Orleans, Indianapolis, and Hamilton public health officials implemented clinics or test centres in community or faith-based sites, and worked to remove barriers to vaccination or testing, such as fees and doctor’s note requirements [33, 36–38]. A Community Ambassadors Program to advance public confidence and trust in the COVID-19 vaccine was additionally noted in Hamilton, Ontario [38]. Further innovations identified in COVID-19 equity included adaptive health communications such as interactive town halls and messaging delivered by revered pioneers in hip hop media (Birmingham, Alabama, and Louisiana, respectively) [33, 39].

Outside the realm of COVID-19, the Allegheny County Health Department in Pennsylvania implemented a community-based chronic disease screening and referral intervention in predominantly Black neighbourhoods in Pittsburgh [40]. To encourage cross-organizational collaboration in service delivery, the Minnesota Department of Health’s Center for Health Equity also developed a directory of resources to simultaneously increase community access to African American Infant Mortality supports across two state counties [41]. Finally, the Bureau of Communicable Disease at the New York City Department of Health and Mental Hygiene forwarded a multi-pronged intervention that offered multiple entry-points for organizational staff to engage in anti-racist pedagogy or praxis [42]. The Bureau used racial identity caucusing, multimedia learning and workshops, social committees, surveillance and data equity training, and hiring, retention, and promotion initiatives to help eliminate its institutional ABR [42].

### Actionable declaration or statement

In the summer of 2020, organizations, institutions, and businesses across the globe were compelled to issue statements condemning ABR, in response to the cumulative evidence on COVID-19 inequities and the uprising of calls to racial justice action following the murder of George Floyd and other victims of police violence in North America. Given this review focuses on SMART initiatives dismantling ABR, any declarations identified needed to include a clear demonstration of implemented and/or planned actions [43]. Eight statements by government institutions declaring ABR as a public health crisis were identified (three in Toronto, one in Peel region (Ontario), one in Ventura County California, and one in Washington State) [44–51]. These declarations made tangible commitments to planned or ongoing action. The commitments included employee training in ABR; public education campaigns; investments in Black-led public health programs; as well as resolutions to invest in or create dedicated organizational positions to address ABR in public health. In Toronto, additional statements included announcements for supplemental funding of Black mental health services and the municipal designation of Black Mental Health Week [44, 46]. At the provincial level, the Alliance for Healthier Communities – a non-governmental network of community health practitioners servicing Ontario – committed to strengthening its Anti-Racism Directorate by devising a systemic ABR strategy and advocating for the allocation of funds for culturally-appropriate supports in communities [50]. Finally, the American Public Health Association detailed intersectoral actions to advance solutions against ABR, such as research and economic investments in structural place-based interventions, and lobbying for intergovernmental policy change in anti-racism [51].

### Budget allocation or investments

Records demonstrating funding allocations and/or investments (*n* = 8) indicated that this type of action spans multiple government bodies in Canada, with a wide range of mandates including public health, heritage, social development, and natural resources [52–60]. This diversity was reflected in areas of focus, with the largest investments allocated to community-based capacity strengthening activities (e.g., $25 million over 5 years) [53, 54, 57]; anti-racism projects ($15 million to 85 projects across Canada) [58]; and culturally-focused mental health programs ($10 million over 5 years) [52]. The City of Toronto’s allocation of $1.2 million to Black health and communities was guided and supported by a Community Accountability Circle, with the intention of centering Black perspectives in the development of funded programs and goals [61]. In the US context, state-level public health departments focused on funding community-level work such as the health and development of Black youth and families in Wisconsin [37], and promoting COVID-19 vaccines through social media in Washington [60].

### Guidance and recommendations for organizational capacity building

Eight records promoted the ways in which an organization can build its capacity to take action around ABR [46, 61–67]. In other words, these records forwarded ‘how’ to build or strengthen the ability of members within an organization, or those in allied organizations, to take informed ABR action. Two of the included records shared self-assessment and reflection tools to help integrate anti-racist action in broad organizational activities. Integration guidelines were oriented to both external services (e.g., an anti-racist COVID-19 response in community) [46, 61] and internal efforts (e.g., culture change, promotion of anti-oppression practice, and learning) [61]. An example of an internal tool is the City of Toronto’s community conversation guide, which aims to build civil servant facilitation skills for community-based discussions on issues connected to ABR [61]. Additional recommendations forwarded at the City of Toronto included representational hiring and promotions of Black individuals [68].

Other recommendations to strengthen ABR action included internal or inter-departmental systems change and staff capacity building [63–65, 69]; centering the social determinants of health, critical social public health practice, and equity as core operational principles (including ethical data disaggregation) [64–66]; and improving capacity to provide culturally appropriate and responsive care to African American communities [64, 67]. The PAHO also provided guidance to partner nations noting the importance of organizational recognition and appreciation of traditional medicine practiced by Afro-descendants and Indigenous peoples [66].

### Task force

The task forces identified (*n* = 9) ranged by breadth of focus, organization/body, and mandate, and membership was primarily limited to health care providers, researchers, and staff within government departments or professional associations. At the federal and state levels, task forces were formed to lead inter-departmental action on the UN International Decade for People of African Descent [53, 70]; to propose reparations for the descendants of previously enslaved African Americans in California [71, 72]; and to analyze the perpetuation of Black health and economic inequalities in Michigan’s state laws [73]. Simultaneously, the American Psychiatric Association formed the ‘APA Presidential Task Force to Address Structural Racism throughout Psychiatry’ to lead structural changes in how the organization is governed; to increase public awareness of the impacts of anti-Black racism on mental health; and to provide broad education on the Association’s history of scientific racism [74, 75].

In contrast with the above records, the Black Scientists’ Task Force on COVID-19 Vaccine Equity at the City of Toronto centered community engagement and involvement in their work [76]. The task force held several town halls to listen and learn from communities, and those conversations formed the basis of a report of recommendations to the City of Toronto on COVID-19 equity [77]. Additional paid sick leave (10 days) and income support, ethical race-based data collection, and improved mental health supports were among the noted recommendations [77].

### Strategy and action plans

A collection of 25 records containing strategic initiatives or action plans were identified. Records were grouped based on a common collection of planned or implemented interventions to effect change in a given area. Organizational strategies were formulated across municipal jurisdictions like the City of Toronto and Middlesex-London (Ontario), and non-governmental entities like the American Psychological Association (APA) and the Association for Multidisciplinary Education and Research in Substance Use and Addition. Committed strategies included the equitable collection of socio-demographic data and related metrics; equitable data reporting and stewardship practices; knowledge mobilization and translation on racial bias; representative hiring; and the inclusion of perspectives from Black community residents in developing programs or policy [78–85]. The APA also formed a task force to develop science-based recommendations to mitigate police violence against Black people as part of its action plan [75]. Further, the City of Toronto, for example, implemented advisory bodies and accountability circles to strengthen organizational responsibility to the desired outcomes of their multi-year Action Plan to Confront Anti-Black Racism (ACABR) [79, 83, 86–88]. A three-year update of the ACABR revealed 60% of recommended actions have been implemented since 2018, with notable culture shifts in employee training, Black representation, and community engagement [87]. Alongside community engagement, data collection was recognized as an important organizational approach for building accountability, tracking outcomes, and reducing inequities in multiple areas of Black health.

In strategies geared toward the public, jurisdictions like the cities of Chicago and Toronto indicated responsiveness to Black health disparities in COVID-19 by implementing needs-based mobile testing, disaggregated COVID-19 data collection measures, and outreach [62, 89]. When not attuned to COVID-19, various levels of government developed strategies to alleviate socioeconomic and political inequalities in Black health. Housing quality and affordability; food insecurity and sovereignty; disproportionate policing and profiling; youth welfare and educational inequities; cultural place-making and environmental justice; and employment and business development, among others, were all noted priorities in Joe Biden’s Action Plan for Black Americans, the New Jersey Department of Children and Families, the City of Toronto’s ACABR, and the City of Toronto’s Black Food Sovereignty Plan [86–88, 90–96]. The latter commits to an ‘iterative co-design and implementation process’ with Black food leaders to foster systems change and ‘culturally-rooted’ food sovereignty for Black Torontonians [94].

Finally, intercultural recognition for diverse therapeutic systems and ways of knowing in the African diaspora, and the policy approaches required to support them, were noted priorities in the action plans of Pan-American organizations like the PAHO and the Inter-American Commission on Human Rights [97, 98].

### Legislation and mandates

Seven records detailed planned or implemented legislative change to address key determinants of Black health inequities. Justice and culturally safe health care were two principal areas of reform. For example, Canadian lawmakers proposed amendments to the Canadian Criminal Code and the Controlled Drugs and Substances Act that would repeal mandatory sentencing for simple drug possession and minimum penalties for imprisonment – conditions that have historically disadvantaged Black and Indigenous peoples [99]. In the US, states like New York and Minnesota passed legislation to outlaw officer chokeholds and aggressive restraint; to enforce reporting on fatal or violent civilian encounters with police; to collect race and demographic data in all low-level offenses; and ordain de-escalation training among enforcement officers [100, 101].

In the provision of health care, legislative changes coalesced around mental health supports for communities with a particular focus on maternal health (i.e., Black Maternal Health Momnibus Act of 2020); provider education on culturally-responsive services (e.g., Strengthening Mental Health Supports for Black, Indigenous, and People of Color Communities Act); and enhanced federal funding for Black mental health programs with an intergenerational focus (e.g., Pursuing Equity in Mental Health Act) [102–104]. Finally, the state of California implemented a resolution mandating the systematic integration of racial equity across the policies of all state sectors such as housing, transportation, and land use planning, among others [105].

## Discussion

**Fig. 3:**
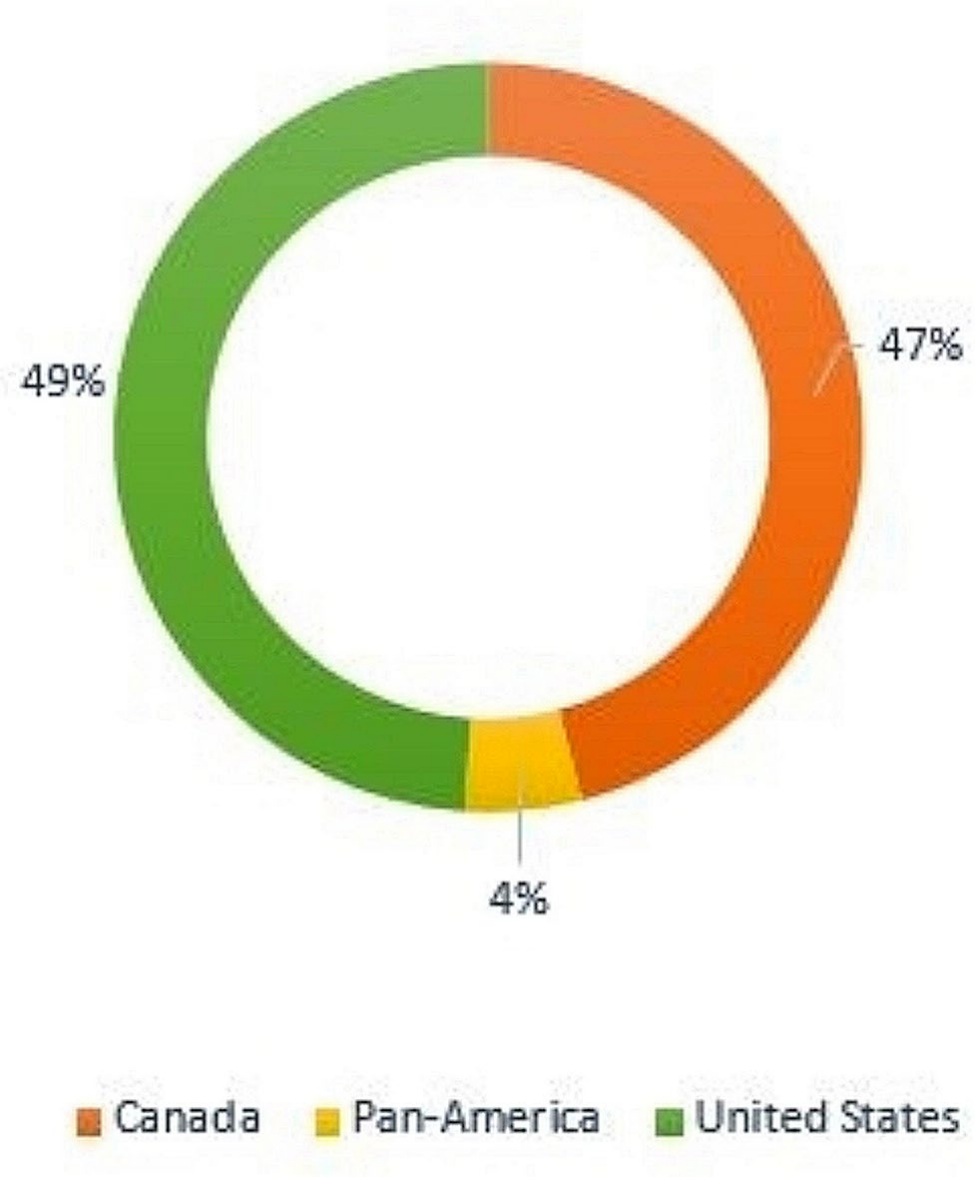
Broad jurisdictions

**Fig. 4:**
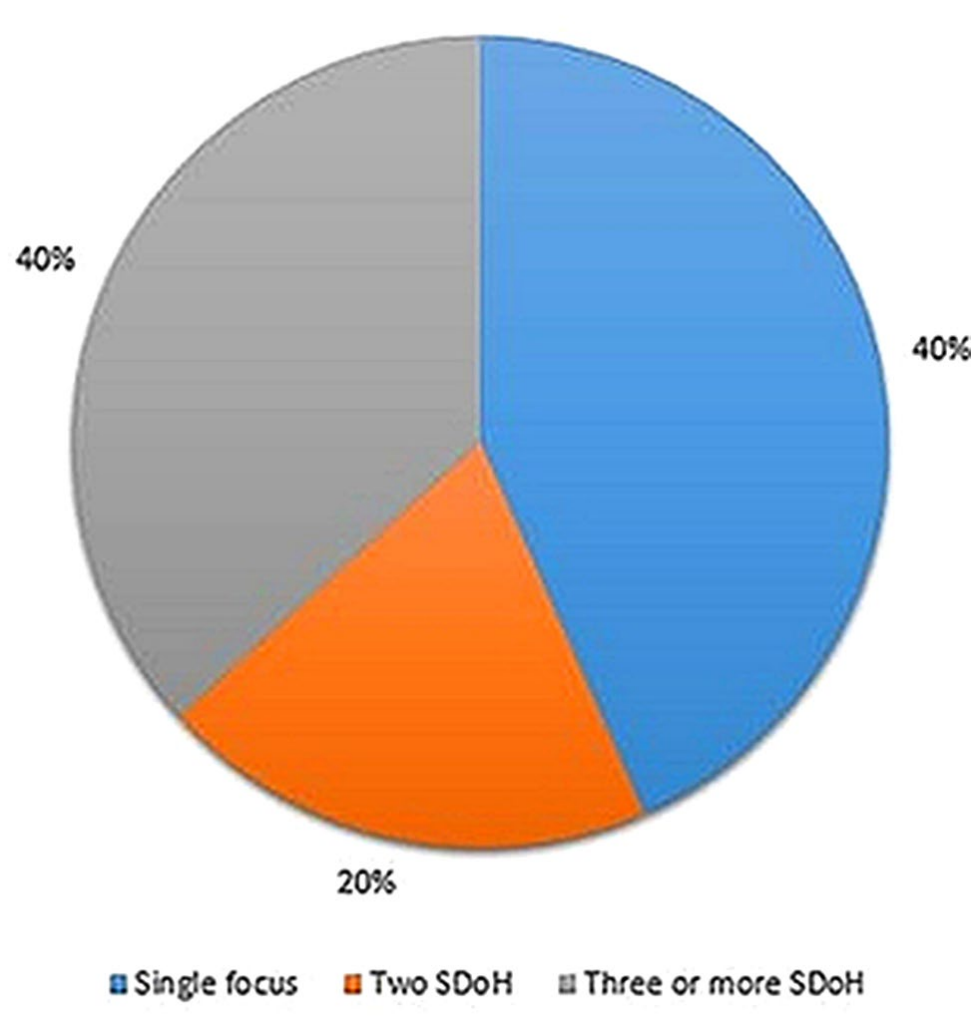
Distribution of actions found by number of foci

Seventy-five articles were included in this review, suggesting that ABR emerged as a public health priority. Strategies and action plans to address structural ABR were the most common types of initiatives observed (*n* = 21), followed by programs or interventions (*n* = 16), budget allocations or investments (*n* = 8), task forces (*n* = 7), guidance and recommendations for organizational capacity (*n* = 8), action-oriented declarations of ABR as a public health crisis (*n* = 8), and legislation and mandates (*n* = 7). The majority of records were captured from Canada (47%) while 49% were of US origin and 4% of records were of Pan-American relevance (Fig. 3). Many actions held a primary or component focus on health, such as equity-driven responses to the COVID-19 pandemic. Outside of COVID-19, health-centric actions aimed to improve health care access, prevent and reduce chronic disease burden, and transform organizational culture to produce more equitable public health services (e.g., establishing representative hiring and mandating anti-racism training). Important legislative change occurred in health (e.g., mental health, maternal health) but transcended health alone (e.g., police accountability and amendments to state and federal criminal justice legislation). Some jurisdictions also led action and/or investments in intersecting determinants of health, such as child and youth development, community services, employment and income supports, policing and justice, and community asset development [88, 89]. It is worth noting that overall, the actions identified were largely intersectoral, multi-faceted, and integrated across the social determinants of health (SDoH) (Fig. 4; Table 1). Further, 20% of records independently targeted two foci, while 40% targeted three or more. The cross-cutting nature of measures to address ABR in this review affirm racism as a multi-issue phenomenon; i.e., structural racism is not only present within systems, but is supported and reinforced across multiple societal systems in reciprocal ways [106]. This inextricable linkage between social systems subsequently challenges any separation of health disparities from the conditions in which they occur and, the institutions accountable for creating those conditions.

### Strengths and limitations

A strength of this review is the focus on completed, in-progress, or committed actions. This operational focus was intended to capture promising practices and contribute to the feasibility of interventions across Canadian and US jurisdictions [107]. The time interval for which initiatives were selected (2019–2022) also captures public health action undertaken on two concurrent and interlocking pandemics – COVID-19 and systemic ABR. While ABR long preceded COVID-19 and is a persisting legacy rooted in the experience and history of enslavement, this review highlights how public opinion and media attention can have direct impacts on the visibility and prioritization of public health issues. More critically, it elucidates the disparate and extreme circumstances required to drive mass action (e.g., police brutality), and whose perception of these events matters in driving these actions forward.

However, this review did not present detailed analyses of the types of public health institutions uncovered in the literature due to the absence of sufficient details, particularly in cases of multi-sectoral partnerships and task forces. This limits our understanding of the organizational context surrounding efforts to dismantle ABR, and is an important consideration for future research. Further, institutional buy-in is but one of several critical drivers of change toward more equitable outcomes and committed or implemented action does not directly infer how institutional actions have benefitted Black communities in meaningful ways. Despite the breadth of actions identified in this review, most of the records did not consistently report the extent or nature of community involvement in the development, implementation, or evaluation of the intervention. Additional shortcomings include an apparent lack of accountability mechanisms in monitoring, reporting, and evaluation activities; cautions against data weaponization and undue surveillance in disaggregated approaches; limited consideration for intersectional violence/identities and decolonial solidarity with Indigenous peoples; and the integration of narrative or qualitative data provided by communities in informing action [108]. While some legislative change was noted in this review (e.g., justice, maternal health) broader opportunities to address upstream determinants across sectors also remain.

Finally, the dearth of actions in Canadian provinces west of Ontario; a sizeable representation of initiatives implemented in Toronto; a methodological exclusion of education initiatives in hospitals, health schools, and various fields of primary and secondary care; and an exclusion of anti-racism initiatives by academic journals are additional limitations. While incidental, a skewed geographic representation may set a good example for metropolitan jurisdictions with similar diverse populations but reduce the relevance of interventions to rural, less socio-demographically diverse contexts. The authors also acknowledge that knowledge production and mobilization in allied fields can have downstream impacts on the public health landscape.

### Implications

A discernable consequence of COVID-19 was the magnified intersectoral awareness for social and politically-driven health inequities that long preceded the pandemic. This research affirms this shift and the concordant movement in public health to mobilize people, data, and resources towards more just outcomes. This review further emphasizes the need for an integrated SDoH lens in public health that recognizes racism as an organized social system of inequality that disempowers certain members of society through differential access to power, social status, money, and privilege [109].

However, this review’s focus on public health practice and governance inadvertently sidelines more participatory blueprints for change and the radical modes of organizing led by communities. It also bears to question whether the depth of anti-oppressive praxis can be meaningfully embodied in public health institutions that historically gained legitimization through experimental trial and error benefitting certain populations over others [110]. Forced sterilization, unethical vaccination trials, and stigmatizing management of crises like HIV/AIDs are notable examples [108, 111–113]. In the North American context, the exclusion and exploitation of Black and Indigenous communities was largely informed by social, medical, and political hegemonies that veiled white supremacist and eugenicist approaches as scientifically necessary, neutral, or sound [114–116]. As recognition of these histories grows across sectors, the widespread adoption of corporate EDI entities has become equated with the anti-racist approach needed for transformative change. Yet, without transformations in knowledge, power, and decision-making hierarchies, and the integration of the unique needs of geographical or identity-based communities in interventions, inequities are likely to be sustained. Further, investments in community assets should be cautioned against the increasing privatization of social services, the neoliberal responsibilization of communities for their marginalization, and other upstream political factors that render funding precarious, conditional, or funnelled selectively toward ‘priority’ areas [117, 118]. Put otherwise, redistributive or ‘liberatory’ interventions may be (un)intentionally delimited to the priorities of dominant interest groups and public health ‘professionals’ [119]. In praxes rooted in CRT, the perspectives of socially marginalized groups should constitute the main axis by which all discourse and decisions take place – the principle of “centering the margins” [120].

As broad work in anti-racism continues, public health action that is sincere in its quest to upend the status quo will thus become more important than ever. Recognition of ABR as a public health crisis alone cannot challenge harmful racial hierarchies, and the interventions showcased in this review are but a starting point for the gaps identified in culturally safe outcome evaluation and community leadership. Participatory engagement of stakeholders, through multiple intervention arms and sectors, who are mutually informed and committed to an ethic of transformation (and simultaneously, refusal to engage in or with, exclusionary systems), are important practices to bridge gaps [120]. Public health institutions should further mobilize action on enduring infrastructure and legislative change that would foster permanent distributive and procedural justice across contexts.

## Conclusion

This scoping review provides an important foundation in spurring continued public health accountability to principles of social justice. Public health must engage critical, transformative actions that transcend passive recognition of racism as an issue and instead take after more radical modes of change. Simultaneously, institutions should respect and give space to, without co-optation, the far-reaching resistance and networks of care that members of Black communities have long organized for themselves in the quest for liberation. Public health plays a central role in maintaining the momentum of racial health justice at the forefront of public consciousness; and while discourses around COVID-19 pandemic “recovery” risk archiving calls for structural change and leaving communities behind, establishing the ‘new normal’ should be a caution for all to consider by whom, and for whom normalcy is defined.

## Data Availability

The data used and/or analyzed during this study are available from the corresponding author on reasonable request.

## Declarations

### Authors information

LR identifies as a Black, cisgender woman and settler with Malagasy ancestry whose public health practice is informed by the complexities of her privilege and marginalization. CBA identities as a cisgender Arab woman with Levantine/Mashreq and Irish ancestry. MS identifies as a white-European woman with Italian ancestry. SA is a Black Muslim woman whose work and scholarship within public health is rooted in her lived experiences and intersectional identities. TW identifies as a cisgender South Asian Muslim woman whose public health work is informed by her living experiences.

## Abbreviations

ABR: Anti-Black racism
ACABR: Action Plan to Confront Anti-Black Racism
APA: American Psychological Association
CRT: Critical Race Theory
EDI: Equity, diversity, and inclusion
PAHO: Pan American Health Organization
PHO: Public Health Ontario
SDoH: Social Determinants of Health
